# The therapeutic efficacy of resveratrol for acute lung injury—A meta−analysis of preclinical trials

**DOI:** 10.3389/fphar.2022.963245

**Published:** 2022-08-25

**Authors:** Yin Tang, Wenqiao Fu, Ke Wei, Ling Liu, Siqi Wu, Wenjing Tang

**Affiliations:** ^1^ Department of Anesthesiology, The First Affiliated Hospital of Chongqing Medical University, Chongqing, China; ^2^ Department of Neurosurgery, The First Affiliated Hospital of Chongqing Medical University, Chongqing, China

**Keywords:** resveratrol, acute lung injury, acute respiratory distress syndrome, animal models, meta−analysis

## Abstract

**Background:** Resveratrol (RES) has a protective effect on acute lung injury (ALI) or acute respiratory distress syndrome (ARDS). Our purpose was to conduct a meta−analysis to investigate the efficacy of RES for ALI/ARDS in animal models.

**Methods:** PubMed, EMBASE and Web of Science were searched to screen relevant preclinical trials. The standardized mean difference (SMD) was used to compare the lung injury score, lung wet−dry weight ratio (W/D ratio), tumor necrosis factor−α (TNF−α), interleukin−1β (IL−1β), IL−6, IL−10, the number of neutrophils in bronchoalveolar lavage fluid (BALF) and the total protein in BALF between the treatment and control groups. SYRCLE’s risk of bias tool was used for quality assessment.

**Results:** A total of 17 studies published from 2005 to 2021 were included in our study to calculate the SMD with corresponding confidence interval (CI). As compared with controls, RES significantly decreased the lung injury score (SMD −2.06; 95% CI −2.77, −1.35; *p* < 0.00001) and W/D ratio (SMD −1.92; 95% CI −2.62, −1.22; *p* < 0.00001). RES also reduced the number of neutrophils in BALF (SMD −3.03; 95% CI −3.83, −2.24; *p* < 0.00001) and the total protein in BALF (SMD −5.59; 95% CI −10.10, −1.08; *p* = 0.02). Furthermore, RES was found to downregulate proinflammatory mediators such as TNF−α (SMD −2.02; 95% CI −3.09, −0.95; *p* = 0.0002), IL−1β (SMD −2.51; 95% CI −4.00, −1.02; *p* = 0.001) and IL−6 (SMD −2.26; 95% CI −3.49, −1.04; *p* = 0.0003). But RES had little effect on the anti−inflammatory mediators such as IL−10 (SMD 2.80; 95% CI −0.04, 5.63; *p* = 0.05). Sensitivity analysis and stratified analysis were performed for the outcome indicators with heterogeneity.

**Conclusion:** RES treatment is effective on reducing the severity of ALI. However, more animal studies and human trials are needed for further investigation. Our study may provide a reference for preclinical and clinical studies in the future to some extent.

## Introduction

ALI/ARDS is a clinically critical illness caused by severe infection and trauma, and its pathological manifestations include acute diffuse lung inflammation, alveolar damage, increased pulmonary capillary permeability, hemorrhage, pulmonary interstitial edema and massive inflammatory cell infiltration (McCallister et al., 2009). In clinical practice, ALI presents with significant respiratory distress, refractory hypoxemia and a progressive decline in lung compliance (Xiao et al., 2011). ALI/ARDS are associated with high morbidity and mortality rates, and its therapeutic effects are often unsatisfactory (Butt et al., 2016). To date, there has been no effective drug therapy for ALI/ARDS.

Resveratrol (3,5,4′−trihydroxy trans−stilbene) was found in different fruits such as grapes, raspberries, mulberries, blueberries and groundnuts (Walle, 2011). Previous studies have shown that RES has antioxidant, anti−inflammatory (Bo et al., 2013), anticancer (Xiao et al., 2018), antidiabetic and cardiovascular−protective effects (Singh et al., 2019; Ahmadi et al., 2021; Meng et al., 2021). RES has been proved to have protective effects on ALI in preclinical studies during recent years (Jiang et al., 2016; Wang et al., 2021). However, it is difficult to evaluate the overall treatment effect due to methodological differences among studies. Up to now, no systematic review or meta−analysis has been conducted to evaluate the quality and synthesize evidences of preclinical studies on the effects of RES on ALI. Furthermore, most studies simulated ALI/ARDS models by cecal ligation and perforation (CLP) or intratracheal instillation of lipopolysaccharide (LPS) (Chimenti et al., 2020). Therefore, preclinical animal studies on ALI may provide some reference for the clinical treatment of ALI/ARDS. We will investigate the efficacy of RES for ALI/ARDS to evaluate whether it can lower lung injury severity via a meta−analysis of animal models.

## Materials and methods

### Search strategy

The systematic review and meta−analysis were conducted by following the Preferred Reporting Items for Systematic Reviews and Meta−Analysis (PRISMA) guidelines (Moher et al., 2009). PubMed, EMBASE and Web of Science (up to 12 May 2022) were searched to screen relevant preclinical trials about the protective effects of RES in animal ALI models. Our search designations were “[Resveratrol (Title/Abstract)] AND [Acute lung injury (Title/Abstract) OR Acute respiratory distress syndrome (Title/Abstract)]”, as shown in Supplementary File S1. The publication language was limited to English.

### Inclusion and exclusion criteria

The inclusion criteria were set up based on the PICOS−scheme (population, intervention, control, outcome and study design). Published studies were included if they met the following criteria: 1) ALI animal model; 2) setting up the RES experimental group; 3) setting a control group with placebo; 4) the effectiveness of RES on the ALI animal models were measured; 5) results were expressed or could be converted to mean and standard deviation; 6) Other criteria: published in English.

The exclusion criteria were as follows: 1) review articles, letters and case reports; 2) repeated publications and abstracts without full text; 3) not reporting the number of animals in each group; 4) animals treated with RES analogues; 5) studies that only tested the effects of RES combined with other chemicals or drugs (such as nanoparticles).

### Data collection

All of the data were extracted independently by two reviewers (Tang and Fu). Any disagreements were resolved by consensus with a third reviewer (Wei). The information collected from each study included the following details: 1) author and year of publication; 2) rodent models, including species, gender, age and modeling methods; 3) anesthetic agents and route of administration; 4) RES treatment, including intervention dosage (initial and total dosage), time point of treatment and route of administration; 5) assessment time; 6) outcome measurement; 7) methodological quality score. If a study comprised multiple experimental groups distinguished by different dosage, time point of treatment and assessment time that were compared with the control groups, these experimental groups would be considered as independent comparisons. For continuous variables, mean and standard deviation were used for statistical calculations. For graphical data, the GetData Graph Digitizer software (version 2.25) would be used to measure values for mean and standard deviation from highly magnified images. For standard deviation without direct report, we would calculate it by multiplying the reported standard error of mean (SEM) by the square root of the group size.

### Quality assessment

Two reviewers (Tang and Fu) independently evaluated the quality of each study according to the SYRCLE’s risk of bias tool (an adaptation of the Cochrane Risk of Bias Tool) (Hooijmans et al., 2014). Any disagreements were resolved by consensus with a third reviewer (Wei).

### Statistical analysis

Our outcomes included lung injury score, W/D ratio, TNF−α, IL−1β, IL−6, IL−10, the number of neutrophils in BALF and the total protein in BALF. We performed statistical analysis using Review Manager (RevMan) 5.4 software (Cochrane Library, London, United Kingdom) and STATA 12 software (StataCorp, College Station, TX, United States). The SMD was used to compare the effect of RES on ALI between the treatment and control groups (Zeng et al., 2021). Heterogeneity among studies was tested by I−squared (I^2^) statistic. I^2^ < 50% indicated low heterogeneity and the fixed−effects model was used, while I^2^ ≥ 50% indicated high heterogeneity and the random−effects model was used (DerSimonian and Laird, 1986; Vetter, 2019). Sensitivity analysis was performed by excluding one study at a time (Zeng et al., 2021). We conducted a stratified meta−analysis to clarify the impact of methodological differences such as animal race, animal gender, methods to induce ALI model, time point of RES treatment, RES treatment route, number of doses and total dosage of RES. A funnel plot was used to check publication bias. Asymmetry was assessed with Egger’s test and the trim−and−fill method (Egger et al., 1997). *p* < 0.05 was considered statistically significant.

## Results

### Study selection

**FIGURE 1:**
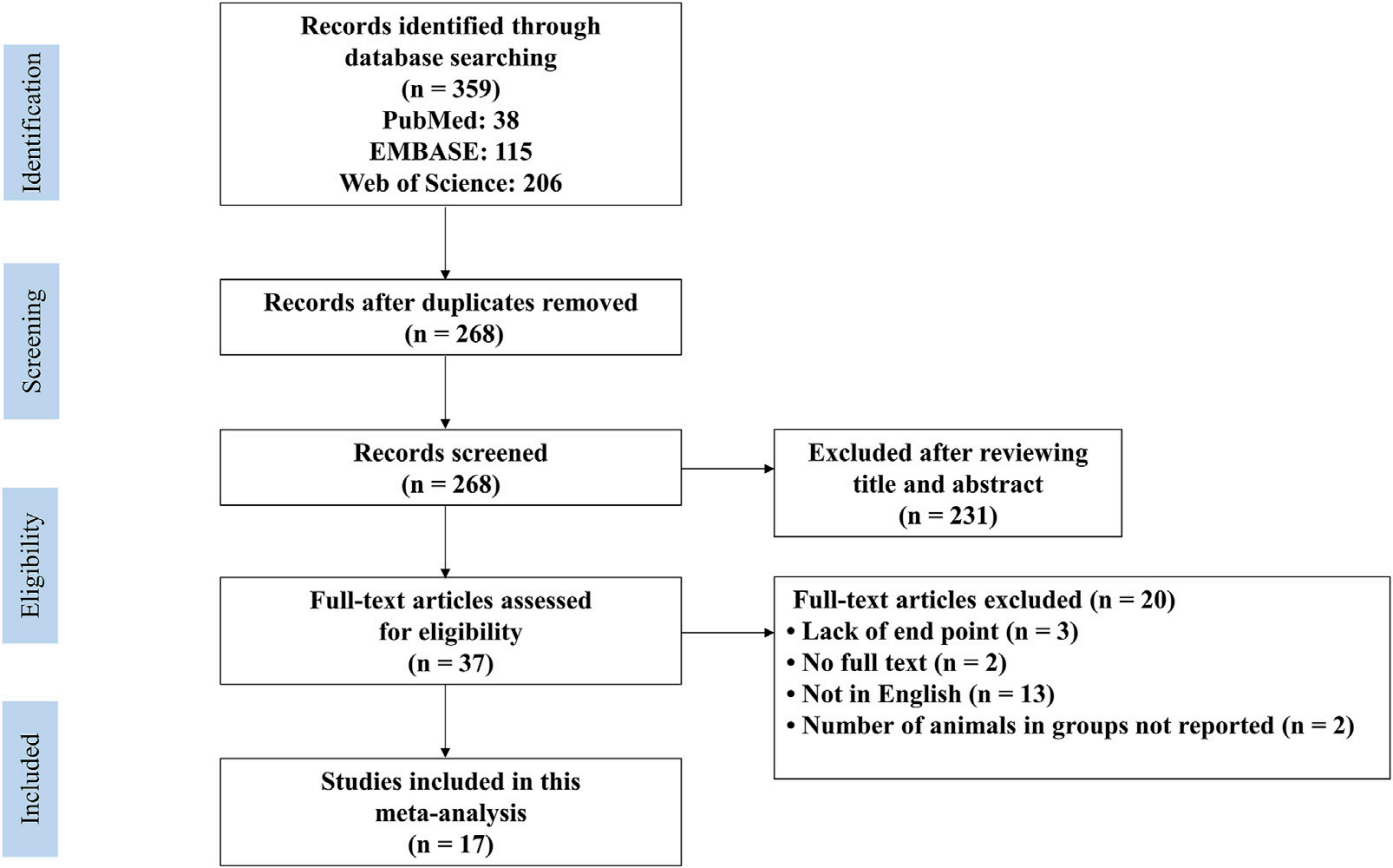
PRISMA flow diagram for review and selection process of studies included in meta−analysis of RES in rodent models of ALI.

The flow diagram of the literature search process is shown in Figure 1. After a comprehensive search, we identified 38 articles in PubMed, 115 articles in EMBASE and 206 articles in Web of Science. After removing duplicates, we screened 268 articles by abstract and/or title, excluding 231 irrelevant records. Then, we retrieved the full text of the remaining 37 records. Among them, 20 records were excluded due to abstracts without full text, not in English, not reporting the number of animals in each group and no adequate outcomes. Finally, we included 17 articles published from 2005 to 2021 (Meng et al., 2005; Sha et al., 2009; Cao et al., 2011; Qi et al., 2012; Rieder et al., 2012; Zhang H.−X. et al., 2014; Zhang Z. et al., 2014; Liu et al., 2015; Jiang et al., 2016; Alghetaa et al., 2018; Wang et al., 2018; Yang et al., 2018; Hu et al., 2019; Tsai et al., 2019; Guo et al., 2021; Jiang et al., 2021; Wang et al., 2021).

### Study characteristics

The animal models were established in C57BL/6 mice (*n* = 7), Sprague–Dawley (SD) rats (*n* = 5), Wistar rats (*n* = 1), C3H/HeJ mice (*n* = 1), BALB/c mice (*n* = 1) and ICR mice (*n* = 2). These studies involved male animals (*n* = 12), female animals (*n* = 4) and one study did not report the gender of animals. Adult animals were used in all studies. Further, these studies chose cecal ligation and puncture (CLP) (*n* = 3), intraperitoneal (i.p.) injection of LPS (*n* = 4), intratracheal (i.t.) instillation of LPS (*n* = 5), administration of staphylococcal enterotoxin B (SEB) (*n* = 2), severe acute pancreatitis (SAP) (*n* = 2) and traumatic spinal cord injury (*n* = 1) to induce the ALI model. In all of them, RES was administered before ALI (*n* = 9), after ALI (*n* = 7) or before and after ALI (*n* = 1). RES was administered by i. p. (*n* = 9), intragastric administration (*n* = 6), injection through the vena dorsalis penis (*n* = 1) and one study did not report the way of administration. And single dose (*n* = 12) or multiple doses (*n* = 5) of RES were used in all studies. The detailed characteristics of the included studies are presented in Table1.

**TABLE 1:** Characteristics of the included studies.

Author, year	Animal, gender	Age	Anesthetic drug	Route	ALI model	Initial dosage	Total dosage	Treatment point	Route	Assessment time	Outcome measurement
Wang, 2021	C57/BL6 mice, Male	Adult	sodium pentobarbital	i.p.	CLP	40 mg/kg	40 mg/kg	after ALI	i.p.	24 h	Lung Injury Score, TNF−α, IL−1β, IL−6
Jiang, 2021	SD rats, Male	Adult	sodium pentobarbital	i.p.	LPS i.p.	0.5 mg/kg	0.5 mg/kg	before ALI	intragastric administration	12 h	Lung Injury Score, W/D, TNF−α, IL−1β, IL−6, Total protein levels in BALF
Guo, 2021	C57/BL6 mice, Female	Adult	sodium pentobarbital	i.p.	LPS i.t.	100 mg/kg	700 mg/kg	before ALI	intragastric administration	24 h	Lung Injury Score, W/D, TNF−α, IL−6, IL−10, Total protein levels in BALF
Tsai, 2019	C57/BL6 mice, Male	Adult	isoflurane	inhalation	LPS i.t.	100 mg/kg	100 mg/kg	before ALI	i.p.	6 h	W/D
Hu, 2019	C57/BL6 mice, Male	Adult	pentobarbital	i.p.	LPS i.t.	30 mg/kg	60 mg/kg	after ALI	i.p.	48 h	Lung Injury Score, IL−1β, Number of neutrophils in BALF
Yang, 2018	C57/BL6 mice, Male	Adult	NR	NR	CLP	40 mg/kg	40 mg/kg	after ALI	i.p.	24 h	Lung Injury Score, TNF−α, IL−1β, IL−6
Wang, 2018	Wistar rats, Male	Adult	chloral hydrate	i.p.	CLP	30 mg/kg	30 mg/kg	after ALI	i.p.	24 h	Lung Injury Score, W/D, IL−10
Alghetaa, 2018	C3H/HeJ mice, Female	Adult	NR	NR	SEB	100 mg/kg	200 mg/kg	before ALI	intragastric administration	3h, 48 h	TNF−α, IL−6
Jiang, 2016	C57/BL6 mice, Male	Adult	NR	NR	LPS i.t.	30 mg/kg	30 mg/kg	before ALI	i.p.	6 h	Lung Injury Score, W/D, IL−1β, Number of neutrophils in BALF
Liu, 2015	SD rats, Female	Adult	chloral hydrate	i.p.	Trauma	100 mg/kg	100 mg/kg	after ALI	i.p.	12 h	Lung Injury Score, W/D, TNF−α, IL−6, IL−10
Zhang (1), 2014	BALB/c mice, NR	Adult	sodium pentobarbital	i.p.	LPS i.t.	5 mg/kg	5 mg/kg	before ALI	intragastric administration	12 h,24 h	W/D, IL−6, Number of neutrophils in BALF
						45 mg/kg	45 mg/kg				
Zhang (2), 2014	ICR mice, Male	Adult	ketamine and xylazine	NR	LPS i.p.	0.3 mg/kg	0.3 mg/kg	before ALI	NR	24 h	Lung Injury Score
Rieder, 2012	C57/BL6 mice, Female	Adult	NR	NR	SEB	100 mg/kg	300 mg/kg	before ALI	intragastric administration	48 h	IL−6
						100 mg/kg	200 mg/kg	after ALI			
Qi, 2012	SD rats, Male	Adult	dentobarbitone	i.p.	LPS i.p.	50 mg/kg	50 mg/kg	before ALI	i.p.	6 h	W/D, TNF−α, IL−6, Total protein levels in BALF
Cao, 2011	ICR mice, Male	Adult	NR	NR	LPS i.p.	1 mg/kg	3 mg/kg	before ALI	intragastric administration	8 h	W/D
						25 mg/kg	75 mg/kg				
Sha, 2009	SD rats, Male	Adult	ketamine	i.p.	SAP	10 mg/kg	10 mg/kg	after ALI	intragastric administration	3, 6, 12 h	Lung Injury Score
Meng, 2005	SD rats, Male	Adult	ketamine	i.p.	SAP	0.1 mg/kg	0.1 mg/kg	after ALI	i.p.	9 h	W/D

Abbreviations: SD, Sprague–Dawley; NR, not reported; i. t., Intratracheal injection; i. p., Intraperitoneal injection; ALI, acute lung injure; CLP, cecal ligation and puncture; LPS, Lipopolysaccharide; SEB, Staphylococcal enterotoxin B; SAP, severe acute pancreatitis; BALF, bronchoalveolar lavage fluid; h, hour; d, day; W/D, Lung wet–dry weight ratio; TNF–α, Tumor necrosis factor–α; IL–1β, Interleukin–1β; IL–6, Interleukin–6; IL–10, Interleukin–10.

### Quality assessment

The details of the study quality are presented in Table 2. The high score represents the high quality of the methodology in the articles. Most of the studies scored 2 to 6 in our study. All the studies reported the “analogous baseline traits” and there were 10 studies that reported “stochastic distribution”. For the “distribution concealment”, “blinded intervening” and “blinded evaluation of result,” there were no reports in the literature. In addition, all the studies were “selecting outcome recording”. There were 13 studies that reported “random collection for outcome measurement”, and 3 studies with other bias like analysis bias. Besides, none of the studies stated sample size counting.

**TABLE 2:** Methodological quality of studies.

Study, year	1	2	3	4	5	6	7	8	9	10	Score
Wang,2021	※	+	※	※	※	+	※	−	+	※	3
Jiang,2021	+	+	※	+	※	+	※	−	+	※	5
Guo,2021	※	+	※	※	※	※	※	※	+	※	2
Tsai,2019	+	+	※	+	※	+	※	−	+	+	6
Hu,2019	※	+	※	※	※	※	※	※	+	※	2
Yang,2018	※	+	※	※	※	+	※	−	+	※	3
Wang,2018	+	+	※	※	※	+	※	−	+	※	4
Alghetaa,2018	※	+	※	※	※	※	※	−	+	※	2
Jiang,2016	+	+	※	+	※	+	※	−	+	※	5
Liu,2015	+	+	※	+	※	+	※	−	+	※	5
Zhang (1),2014	+	+	※	+	※	※	※	−	+	※	4
Zhang (2),2014	+	+	※	+	※	+	※	−	+	+	6
Rieder,2012	※	+	※	※	※	+	※	−	+	+	4
Qi,2012	+	+	※	※	※	+	※	−	+	※	4
Cao,2011	※	+	※	※	※	+	※	−	+	※	3
Sha,2009	+	+	※	+	※	+	※	−	+	※	5
Meng,2005	+	+	※	+	※	+	※	−	+	※	5

1-stochastic distribution sequence; 2-analogous baseline traits; 3-distribution concealment; 4-stochastic housing; 5-blinded intervening; 6-random collection for outcome measurement; 7-blinded evaluation of result; 8-unfinished outcome data; 9-selecting outcome recording; 10-else sources of bias. +: yes; −: no; ※: unclear.

### Meta−analysis

**FIGURE 2:**
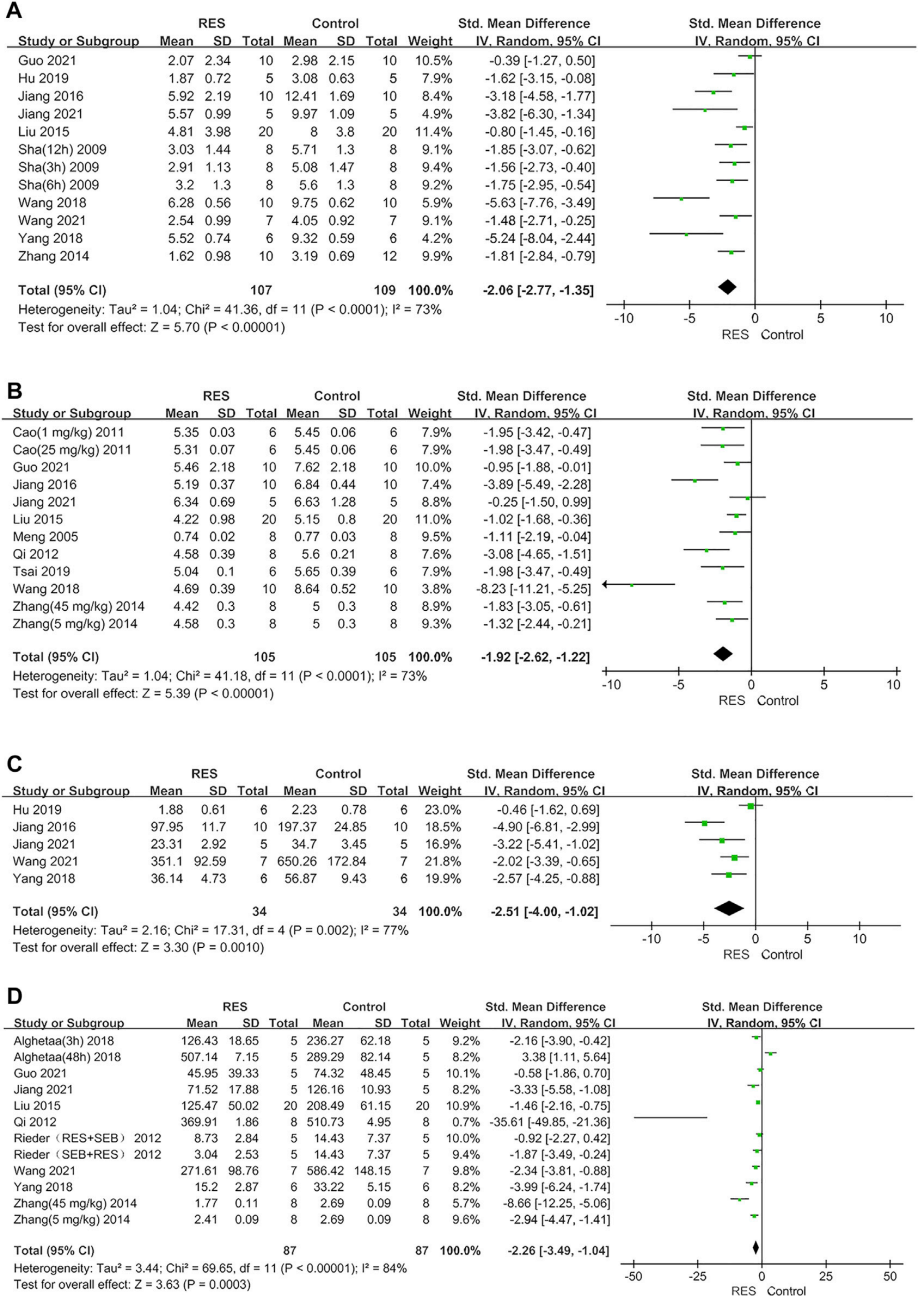
**(A)** Forest plot analyzing the effect of RES treatment of lung injury score. **(B)** Forest plot analyzing the effect of RES treatment of W/D ratio. **(C)** Forest plot analyzing the effect of RES treatment of IL−1β. **(D)** Forest plot analyzing the effect of RES treatment of IL−6.

The RES could significantly reduce the lung injury score by an SMD of −2.06 (95% CI: −2.77, −1.35; *p* < 0.00001, 10 studies, 12 comparisons, Figure 2A), with a statistically significant heterogeneity (I^2^ = 73%; *p* < 0.0001). RES reduced the W/D ratio by an SMD of −1.92 (95% CI: −2.62, −1.22; *p* < 0.00001, 10 studies, 12 comparisons, Figure 2B), with a statistically significant heterogeneity (I^2^ = 73%; *p* < 0.0001). RES downregulated proinflammatory mediators IL−1β by an SMD of −2.51 (95% CI: −4.00, −1.02; *p* = 0.001, 5 studies, Figure 2C), with a statistically significant heterogeneity (I^2^ = 77%; *p* = 0.002). RES downregulated IL−6 by an SMD of −2.26 (95% CI: −3.49, −1.04; *p* = 0.0003, 9 studies, 12 comparisons, Figure 2D), with a statistically significant heterogeneity (I^2^ = 84%; *p* < 0.00001). So, we did further stratified analysis from animal race, animal gender, methods to induce ALI model, time point of RES treatment, RES treatment route, number of doses and total dosage of RES in lung injury score, W/D ratio, IL−1β and IL−6.

**FIGURE 3:**
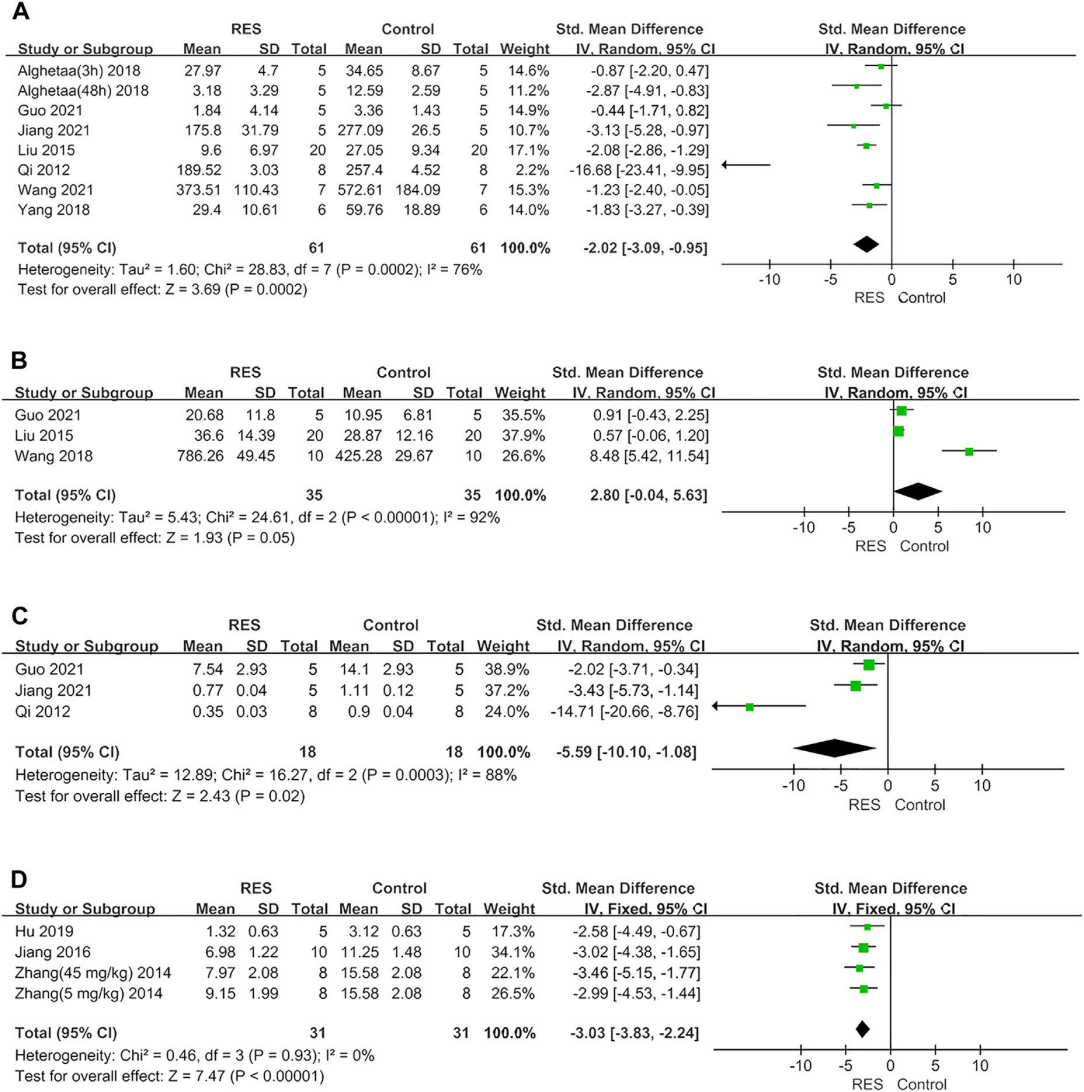
**(A)** Forest plot analyzing the effect of RES treatment of TNF−α. **(B)** Forest plot analyzing the effect of RES treatment of IL−10. **(C)**. Forest plot analyzing the effect of RES treatment of total protein in BALF. **(D)** Forest plot analyzing the effect of RES treatment of number of neutrophils in BALF.

Furthermore, RES was found to downregulate proinflammatory mediators TNF−α by an SMD of −2.02 (95% CI: −3.09, −0.95; *p* = 0.0002, 7 studies, 8 comparisons, Figure 3A), with a statistically significant heterogeneity (I^2^ = 76%; *p* = 0.0002). RES upregulated anti−inflammatory mediators IL−10 by an SMD of 2.80 (95% CI: −0.04, 5.63; *p* = 0.05, 3 studies, Figure 3B), with a statistically significant heterogeneity (I^2^ = 92%; *p* < 0.00001). RES reduced the total protein in BALF by an SMD of −5.59 (95% CI: −10.10, −1.08; *p* = 0.02, 3 studies, Figure 3C), with a statistically significant heterogeneity (I^2^ = 88%; *p* = 0.0003). So, we did further sensitivity analysis in TNF−α, IL−10 and total protein in BALF. The treatment of RES had a favorable effect on the number of neutrophils in BALF by an SMD of −3.03 (95% CI: −3.83, −2.24; *p* < 0.00001, 3 studies, 4 comparisons, Figure 3D), with a low heterogeneity (I^2^ = 0%; *p* = 0.93). Therefore, further stratified analysis was not performed.

### Stratified analysis

For lung injure score, there was no significant difference in estimated effect size among ALI induction methods (*p* = 0.09), the time point of RES treatment (*p* = 0.98) and RES treatment route (*p* = 0.67). Significant differences among subgroups were found in animal race (*p* = 0.0006), animal gender (*p* = 0.001), number of doses (*p* = 0.04) and total dosage of RES (*p* = 0.004). Among them, there was a clear difference in therapeutic effect by the number doses of RES and animal gender. Compared with single dose (SMD −2.33, 95% CI −3.13, −1.53), the effects size of multiple doses (SMD −0.83, 95% CI −1.99, −0.32) was less than single dose. In addition, the effect size was greater in males (SMD −2.42, 95% CI −3.15, −1.69) than in females (SMD −0.66, 95% CI −1.18, −0.14) (Table 3).

**TABLE 3:** Stratified meta−analysis of lung injury score.

**Subgroup**	**Studies**	**SMD (95%CI)**	**Heterogeneity test**		***p***
			***I*** ^***2***^ **(%)**	***p***	
**1. Animal**					
C57BL/6 mice	5	−2.07 (−3.42, −0.72)	78%	0.001	
SD rats	5	−1.57 (−2.29, −0.84)	48%	0.1	
Wistar rats	1	−5.63 (−7.76, −3.49)			
ICR mice	1	−1.81 (−2.84, −0.79)			
					0.006
**2. Gender**					
Male	10	−2.42 (−3.15, −1.69)	60%	0.008	
Female	2	−0.66 (−1.18, −0.14)	0%	0.46	
					0.0001
**3. ALI Model**					
CLP	3	−3.97 (−7.03, −0.91)	86%	0.0009	
LPS i.p.	2	−2.48 (−4.34, −0.63)	53%	0.14	
LPS i.t.	3	−1.66 (−3.38, −0.05)	82%	0.004	
Trauma	1	−0.80 (−1.45, −0.16)	0%	0.95	
SAP	3	−1.71 (−2.40, −1.02)			
					0.09
**4. RES Treatment point**					
after ALI	8	−2.10 (−2.98, −1.21)	73%	0.0005	
before ALI	4	−2.08 (−3.53, −0.62)	80%	0.002	
					0.98
**5. RES Route**					
intragastric administration	2	−1.90 (−5.24, 1.44)	85%	0.01	
i.p.	6	−2.70 (−4.11, −1.29)	84%	0.0001	
vena dorsalis penis	3	−1.71 (−2.40, −1.02)	0%	0.95	
NR	1	−1.81 (−2.84, −0.79)			
					0.67
**6. Number of doses**					
Single dose	10	−2.33 (−3.13, −1.53)	73%	0.0001	
Multiple doses	2	−0.83 (−1.99, −0.32)	46%	0.18	
					0.04
**7.Total dosage of RES**					
<10 mg/kg	2	−2.48 (−4.34, −0.63)	53%	0.14	
10–50 mg/kg	7	−2.61 (−3.60, −1.61)	69%	0.003	
50–200 mg/kg	2	−0.93 (−1.52, −0.33)	0%	0.34	
>200 mg/kg	1	−0.39 (−1.27, 0.50)			
					0.004

For W/D ratio, there was no significant difference in estimated effect size among animal gender (*p* = 0.08), the time point of RES treatment (*p* = 0.42), RES treatment route (*p* = 0.05), number of doses (*p* = 0.23) and total dosage of RES (*p* = 0.05). Significant differences among subgroups were found in animal race (*p* = 0.0005) and ALI induction methods (*p* = 0.0001) (Table 4).

**TABLE 4:** Stratified meta−analysis of lung wet−dry weight ratio.

**Subgroup**	**Studies**	**SMD (95%CI)**	**Heterogeneity test**		***p***
			***I*** ^***2***^ **(%)**	***p***	
**1. Animal**					
C57BL/6 mice	3	−2.18 (−3.87, −0.49)	79%	0.008	
SD rats	4	−1.24 (−2.12, −0.36)	62%	0.05	
Wistar rats	1	−8.23 (−11.21, −5.25)	0%	0.97	
ICR mice	2	−1.96 (−3.01, −0.92)			
					0.0005
**2. Gender**					
Male	7	−2.60 (−3.96, −1.25)	82%	0.00001	
Female	2	−1.00 (−1.54, −0.45)	0%	0.90	
NR	2	−1.55 (−2.38, −0.73)	0%	0.55	
					0.08
**3. ALI Model**					
CLP	1	−8.23 (−11.21, −5.25)			
LPS i.p.	4	−1.75 (−2.95, −0.56)			
LPS i.t.	5	−1.86 (−2.74, −0.97)	64%	0.04	
Trauma	1	−1.02 (−1.68, −0.36)	61%	0.04	
SAP	1	−1.11 (−2.19, −0.04)			
					0.0001
**4. RES Treatment point**					
after ALI	3	−2.81 (−5.15, −0.46)	91%	0.0001	
before ALI	9	−1.80 (−2.47, −1.14)	57%	0.02	
					0.42
**5. RES Route**					
intragastric administration	6	−1.29 (−1.80, −0.77)	9%	0.36	
i.p.	6	−2.80 (−4.21, −1.39)	85%	0.00001	
					0.05
**6. Number of doses**					
Single dose	9	−2.12 (−3.04, −1.19)	80%	0.00001	
Multiple doses	3	−1.92 (−2.62, −1.22)	73%	0.0001	
					0.23
**7.Total dosage of RES**					
<10 mg/kg	4	−1.11 (−1.73, −0.50)	6%	0.37	
10–50 mg/kg	4	−3.89 (−5.89, −1.90)	82%	0.0009	
50–200 mg/kg	3	−1.56 (−2.31, −0.80)	0%	0.53	
>200 mg/kg	1	−0.95 (−1.88, −0.01)			
					0.05

For IL−1β, no significant difference in estimated effect size among animal race (*p* = 0.55), ALI induction methods (*p* = 0.73) and RES treatment route (*p* = 0.55). Significant differences among subgroups were found in time point of RES treatment (*p* = 0.02), number of doses (*p* = 0.003) and total dosage of RES (*p* = 0.01). Among them, there was a clear difference in therapeutic effect by the number doses of RES. The effect size was greater in single dose (SMD −3.06, 95% CI −4.13, −1.82) than in multiple doses (SMD −0.46, 95% CI −1.62, −0.69) (Table 5).

**TABLE 5:** Stratified meta−analysis of IL−1β.

**Subgroup**	**Studies**	**SMD (95%CI)**	**Heterogeneity test**		***p***
			***I*** ^***2***^	***p***	
**1. Animal**					
C57BL/6 mice	4	−2.38 (−4.11, −0.64)	81%	0.001	
SD rats	1	−3.22 (−5.41, −1.02)			
					0.55
**2. ALI Model**					
CLP	2	−2.24 (−3.30, −1.17)	0%	0.62	
LPS i.p.	1	−3.22 (−5.41, −1.02)	93%	0.0001	
LPS i.t.	2	−2.61 (−6.96, 1.74)			
					0.73
**3. RES Treatment point**					
after ALI	3	−1.58 (−2.87, −0.29)	61%	0.08	
before ALI	2	−4.15 (−5.79, −2.51)	22%	0.26	
					0.02
**4. RES Route**					
intragastric administration	1	−3.22 (−5.41, −1.02)			
i.p.	4	−2.38 (−4.11, −0.64)	81%	0.001	
					0.55
**5. Number of doses**					
Single dose	4	−3.06 (−4.13, −1.82)	50%	0.11	
Multiple doses	1	−0.46 (−1.62, −0.69)			
					0.003
**6.Total dosage of RES**					
<10 mg/kg	1	−3.22 (−5.41, −1.02)			
10–50 mg/kg	3	−3.06 (−4.70, −1.42)	66%	0.05	
50–200 mg/kg	1	−0.46 (−1.62, 0.69)			
					0.01

For IL−6, no significant difference in estimated effect size among animal race (*p* = 0.19), ALI induction methods (*p* = 0.16), the time point of RES treatment (*p* = 0.62) and RES treatment route (*p* = 0.27). Significant differences among subgroups were found in animal gender (*p* = 0.03), number of doses (*p* = 0.06) and total dosage of RES (*p* = 0.002). Among them, there was a clear difference in therapeutic effect by the number doses of RES. The effect size was greater in single dose (SMD −3.96, 95% CI −5.85, −2.07) than in multiple doses (SMD −0.57, 95% CI −2.05, 0.91) (Table 6).

**TABLE 6:** Stratified meta−analysis of IL−6.

**Subgroup**	**Studies**	**SMD (95%CI)**	**Heterogeneity test**		***p***
			***I*** ^***2***^ **(%)**	***p***	
**1. Animal**					
C57BL/6 mice	5	−1.73 (−2.76, −0.71)	55%	0.07	
SD rats	3	−5.91 (−11.21, −0.61)	92%	0.00001	
C3H/HeJ mice	2	0.56 (−4.87, 5.98)	93%	0.84	
BALC/c mice	2	−5.56 (−11.14, −0.02)	88%	0.004	
					0.19
**2. Gender**					
Male	4	−4.90 (−8.44, −1.36)	86%	0.0001	
Female	6	−0.81 (−1.89, 0.28)	74%	0.002	
NR	2	−5.56 (−11.14, 0.02)	88%	0.004	
					0.03
**3. ALI Model**					
CLP	2	−2.93 (−4.49, −1.38)	31%	0.23	
LPS i.p.	2	−18.67 (−50.26, 12.92)	95%	0.0001	
LPS i.t.	3	−3.61 (−6.94, −0.27)	90%	0.0001	
Trauma	1	−1.46 (−2.16, −0.75)	82%	0.0007	
SEB	4	−0.52 (−2.55, 1.51)			
					0.16
**4. RES Treatment point**					
after ALI	4	−2.06 (−2.95, −1.17)	41%	0.17	
before ALI	8	−2.65 (−4.82, −0.49)	89%	0.00001	
					0.62
**5. RES Route**					
intragastric administration	8	−1.87 (−3.41, −0.33)	83%	0.00001	
i.p.	4	−3.67 (−6.48, −0.87)	89%	0.00001	
					0.27
**6. Number of doses**					
Single dose	7	−3.96 (−5.85, −2.07)	86%	0.00001	
Multiple doses	5	−0.57 (−2.05, 0.91)	77%	0.002	
					0.006
**7.Total dosage of RES**					
<10 mg/kg	2	−3.06 (−4.33, −1.80)	0%	0.78	
10–50 mg/kg	4	−7.43 (−12.21, −2.65)	90%	0.00001	
50–200 mg/kg	4	−0.72 (−2.52, 1.09)	83%	0.0005	
>200 mg/kg	2	−0.74 (−1.67, 0.18)	0%	0.72	
					0.002

### Sensitivity analysis

**FIGURE 4:**
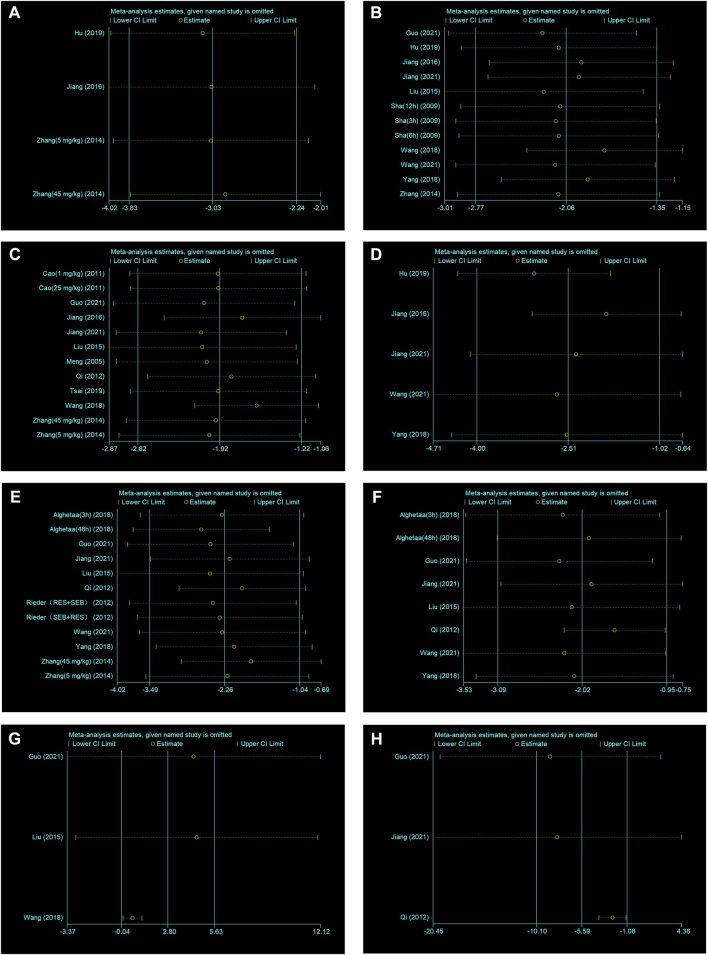
**(A)** Sensitivity analysis of RES treatment of number of neutrophils in BALF. **(B)** Sensitivity analysis of RES treatment of lung injury score. **(C)** Sensitivity analysis of RES treatment of W/D ratio. **(D)** Sensitivity analysis of RES treatment of IL−1β. **(E)** Sensitivity analysis of RES treatment of IL−6. **(F)** Sensitivity analysis of RES treatment of TNF−α. **(G)** Sensitivity analysis of RES treatment of IL−10. **(H)** Sensitivity analysis of RES treatment of total protein in BALF.

**FIGURE 5:**
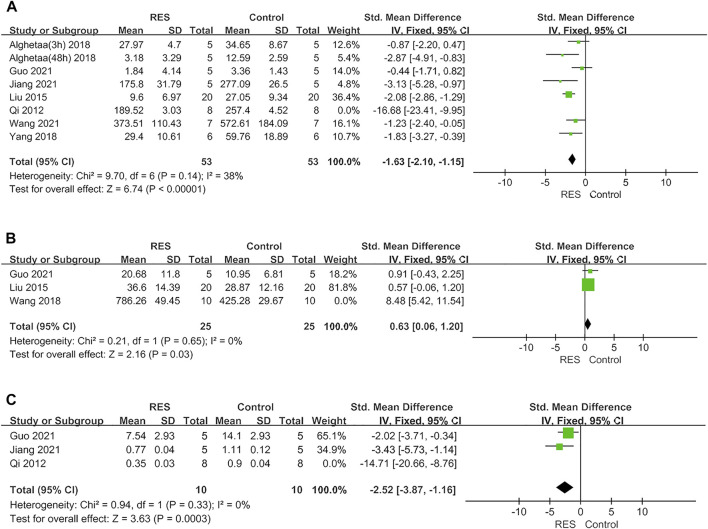
**(A)** Forest plot analyzing the effect of RES treatment of TNF−α after removing studies with a large effect on heterogeneity. **(B)** Forest plot analyzing the effect of RES treatment of IL−10 after removing studies with a large effect on heterogeneity. **(C)** Forest plot analyzing the effect of RES treatment of total protein in BALF after removing studies with a large effect on heterogeneity.

A sensitivity analysis was conducted to evaluate the stability of our results. The number of neutrophils in BALF, lung injury score, W/D ratio, IL−1β and IL−6 were not significantly affected by any study for the pooled SMD (Figures 4A–E−). But TNF−α, IL−10 and total protein in BALF were affected by the pooled SMD (Figures 4F–H−). Meta−analyses were repeated using a fixed−effects model after removing studies with a large effect on heterogeneity (Figures 5A–C−).

### Publication bias

**FIGURE 6:**
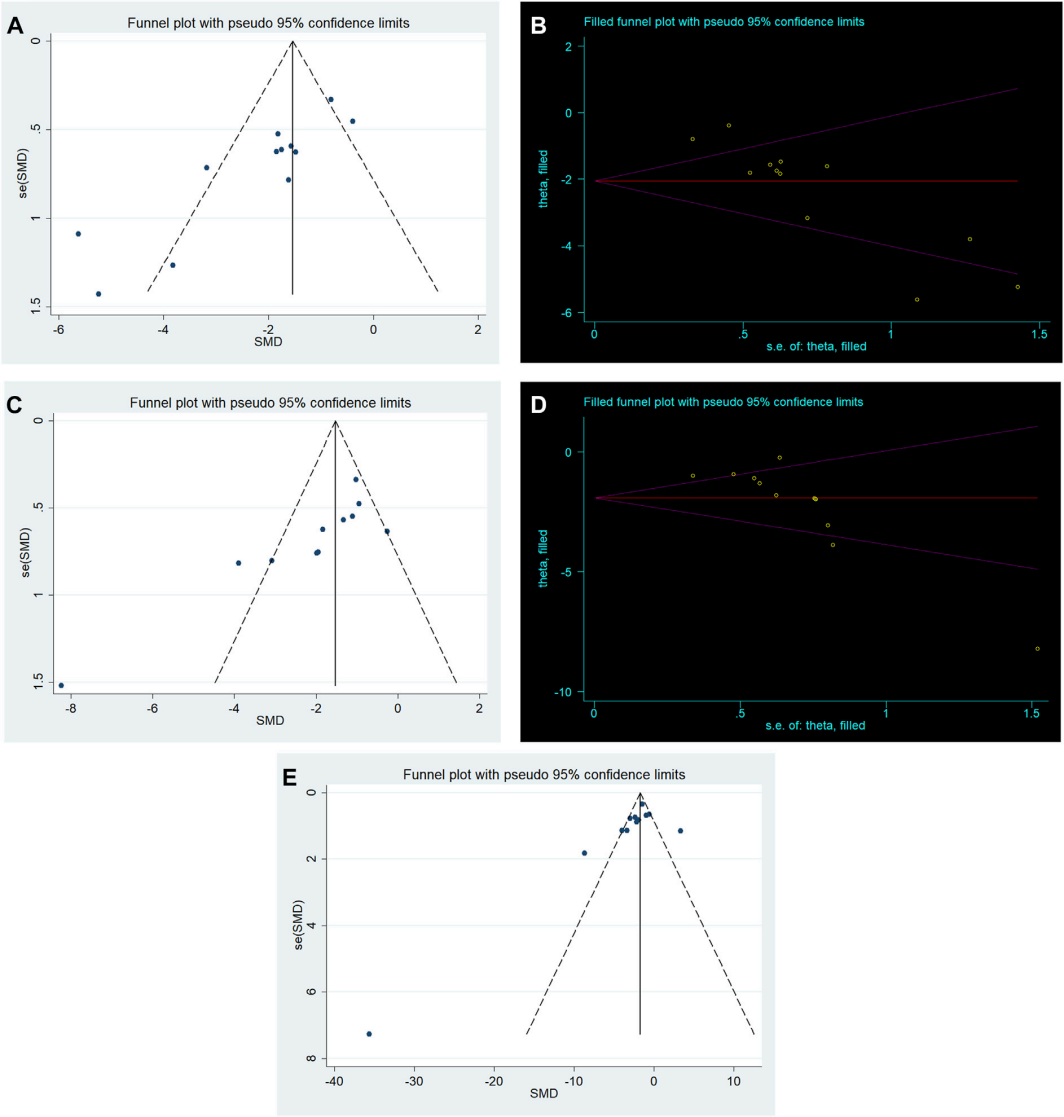
**(A)** Funnel plots for RES treatment of lung injury score. **(B)** Trim−and−fill analysis of RES treatment of lung injury score. **(C)** Funnel plots for RES treatment of W/D ratio. **(D)** Trim−and−fill analysis of RES treatment of W/D ratio. **(E)** Funnel plots for RES treatment of IL−6.

We performed publication bias analysis for outcome measures with more than 10 included studies. Conspicuous publication bias for lung injure score and W/D ratio were suggested by visual inspection of the funnel plot (Figures 6A,B). Then, the Egger’s test confirmed the existence of publication bias in lung injury score (*p* = 0.000) and W/D ratio (*p* = 0.002). Under the circumstances, to estimate the missing studies and recalculate effect estimates, the trim−and−fill analysis was conducted for lung injury score and W/D ratio. The results of lung injure score (SMD −2.059; 95% CI −2.768, −1.351; *p* = 0.000) and W/D ratio (SMD −1.922; 95% CI −2.621, −1.222; *p* = 0.000) were consistent, indicating no “missing” studies (Figures 6D,E). The funnel plot for IL−6 was approximately symmetrical (Figure 6C), and no significant publication bias was confirmed by the result of the Egger’s test (*p* = 0.07).

## Discussion

### Summary of evidence

RES is a promising lung injury protector that has shown beneficial outcomes in animal models of ALI (Jiang et al., 2016; Wang et al., 2021). To our knowledge, no meta−analysis has been conducted to evaluate the efficacy of RES in ALI animal models. Our study demonstrated the anti−inflammatory efficacy of RES treatment in ALI animal models. In our study, the pooled results indicated that RES significantly reduced lung injury score, which is direct evidence that RES can attenuate lung injury severity (Kulkarni et al., 2022). The W/D ratio of the lung is an extensively utilized parameter to assess pulmonary vessel permeability in animal studies, which was demonstrated to be decreased in our study. This reduced ratio indicated that RES can improve lung water clearance (Wang et al., 2020). RES also reduced the number of neutrophils in BALF and the total protein in BALF. Moreover, our study revealed that RES can downregulate the levels of inflammatory factors such as TNF−α, IL−1β and IL−6. But RES had little effect on the IL−10, a well−known anti−inflammatory factor. The results of this meta−analysis in preclinical studies suggest that RES may have potential application value in providing protective effects for clinical ALI patients.

### Possible mechanisms

Over the past few decades, researches have sought for various potential natural compounds to treat ALI, while RES became a popular choice for many studies (Ahmadi et al., 2021). RES contained several functions including mitochondrial protective effect, enhancement of autophagy (Wang et al., 2021), anti−inflammatory (Cao et al., 2011) and anti−apoptotic effect (Sha et al., 2009; Jiang et al., 2021). RES treatment attenuated LPS−induced lung pathological damage, pulmonary edema, oxidative stress (Zhang H.−X. et al., 2014), macrophage infiltration (Yang et al., 2018; Hu et al., 2019) and neutrophil infiltration (Rieder et al., 2012; Zhang Z. et al., 2014; Tsai et al., 2019). These effects largely mitigated lung injury and improved its prognosis. The mechanisms involved in RES may include inhibition of nuclear factor−κB p65 nuclear translocation (Cao et al., 2011; Rieder et al., 2012; Liu et al., 2015; Jiang et al., 2016; Yang et al., 2018), inhibition of myd88−dependent Toll−like receptor 4 signaling pathway (Zhang Z. et al., 2014), inhibition of long noncoding RNA expression (Jiang et al., 2021) and inhibition of NLRP3 inflammasome activation (Jiang et al., 2016). Furthermore, its therapeutic mechanism may also be related to inhibition of the phosphorylation of p38 Mitogen Activated Protein Kinase (MAPK) and Intercellular Adhesion Molecule−1 (ICAM−1) (Qi et al., 2012). These mechanisms provide a reference for future exploration of therapeutic targets for ALI. Therefore, we think that RES is a promising protective candidate in the future, which deserves more researches in the treatment of ALI.

### Interpretation of stratified analysis

In this meta−analysis, RES had significant protective effects in reducing lung injury score, W/D ratio, TNF−α, IL−1β, IL−6, the total protein and number of neutrophils in BALF. But the heterogeneity was statistically significant among lung injury score, W/D ratio, IL−1β and IL−6 groups. Thus, we performed stratified analysis from animal race, animal gender, methods to induce ALI model, time point of RES treatment, RES treatment route, number of doses and total dosage of RES.

### Animal race and gender

First, our stratified meta−analysis of lung injury scores and W/D ratio found that animal race was a source of heterogeneity. The researchers selected C57BL/6 mice, SD rats, Wistar rats and ICR mice for the study. Among them, Wistar rats has the largest effect size, but only one study chose the Wistar rats. A small number of studies might lead to an overestimate on the efficacy of RES. Furthermore, we found that most studies selected C57BL/6 mice and SD rats for their studies. Second, we found that animal gender was a source of heterogeneity through our stratified meta−analysis of lung injury scores and IL−6. We found that most studies selected male animals, which also had larger effect sizes. Previous studies have reported that male mice are more likely to develop ARDS (Chen et al., 2010). However, we can’t confirm that male animals are more suitable for the ALI model due to fewer studies involving female animals. Therefore, normalizing the selection of animal race and gender needs to be done in the future.

## Methods to induce acute lung injury model

Our stratified meta−analysis of W/D ratio found that the source of heterogeneity was related to the methods to induce ALI model. Among them, CLP had the largest effect size, but it didn’t mean that CLP was the most suitable modeling method of ALI. Furthermore, we found that most of the studies selected LPS i. p. And LPS i. t. for study. A previous study found that the ALI model induced by intratracheal instillation of LPS was more effective than the ALI model induced by CLP (Chimenti et al., 2020). However, the severity of lung injury can vary significantly depending on the dose of LPS administered. Therefore, more researches are needed to determine which methods are more suitable for ALI studies in the future.

### Timing, frequency, route and dosage of resveratrol treatment

First, our stratified meta−analysis found that the route of RES treatment was not a source of heterogeneity. Second, the time point of RES treatment was found to be a source of its heterogeneity through stratified meta−analysis of IL−1β. We found that more studies gave RES after ALI modeling, but these included smaller effect sizes. Therefore, we can’t confirm when it is better to give RES. Third, our stratified meta−analysis of lung injure score, IL−1β and IL−6 found that the number of doses and total dosage of RES were a source of heterogeneity. We found that most studies selected single dose, which also had larger effect sizes. In our included studies, the total dosage of RES was 0.1–−700 mg/kg. Among them, most of the studies selected 10–−50 mg/kg for study, which had the largest effect size. Previous studies have shown, increasing the dose of RES can reduce lung injury scores and inflammatory factor levels (Zhang Z. et al., 2014). But high doses may have certain side effects (Shaito et al., 2020). In addition, our study found that studies using 10–−50 mg/kg RES also used a single dose. Therefore, we can conclude that a single dose of RES at 10–−50 mg/kg may have a better therapeutic effect on ALI. However, studies have found poor oral bioavailability of RES (Walle, 2011). In humans, RES has a short initial half−life (8–−14 min for the primary molecule). The plasma half−life of total RES metabolites was 9.2 h and the absorption rate was 75% after oral administration, and the elimination half−life of total RES metabolites was 11.4 h after intravenous injection (Walle et al., 2004; Baur and Sinclair, 2006; Walle, 2011). In SD rats, the elimination half−life of RES analogs was 4.8 h after intravenous injection (Ng et al., 2014). Because the half−life of RES in animals and humans is inconsistent, and the effect of RES on ALI/ARDS has not been clinically performed, we are currently unable to unify the correspondence between the doses used in animal models and those used in humans. The clinical translation of RES in ALI/ARDS requires more time and research. Thus, more researches are needed in the future to determine the timing, frequency, route and dosage of RES treatment.

### Advantages and limitations

In our meta−analysis, we have made great efforts to obtain objective results. First, to provide the most complete evidence, we searched for reports relevant to the field. Then, to reduce potential publication bias of included studies, two reviewers independently assessed and extracted all data, and differences were handled reasonably. Finally, our meta−analysis showed that RES treatment had positive outcomes in ALI animal models. Sensitivity analysis confirmed stable results of the number of neutrophils in BALF, lung injury score, W/D ratio, IL−1β and IL−6, while stratified analysis detected heterogeneity in the result of lung injury score, W/D ratio, IL−1β and IL−6 from methodological differences. To some extent the results suggested positive effect of RES treatment, indicating RES may serve as a new therapeutic strategy for the clinical ALI patients.

There are several limitations in our meta−analysis. 1) Our study only included data published in English and studies that had been published, and some negative results were less likely to be published. Therefore, this meta−analysis may have exaggerated the effect size. 2) Our study included comparatively small number of published studies with a highly significant heterogeneity. Although a further stratified analysis was conducted, the differences among most subgroups were still not significant. These results may be related to insufficient sample size. Therefore, sufficient evidence needs to be provided in the studies with large sample sizes in the future. 3) We did not analyze the timing of outcome assessment. Different time of assessment will also have different results. For example, at 48 h after RES intervention, the inflammatory factor level of TNF−α was found to be lower than that at 3 h. IL−6 levels were found to decrease at 3 h after RES intervention, but increase at 48 h (Alghetaa et al., 2018). Therefore, the evaluation time of RES intervention will affect the outcome of ALI, and more researches are needed in the future to confirm the most appropriate evaluation time.

## Conclusion

Our systematic review and meta−analysis revealed that RES treatment had an improvement in pathology, pulmonary edema and inflammatory markers in ALI animal models. Limitations of the experimental design should be considered when interpreting the results. The results of this study may provide a reference for preclinical and clinical studies in the future to some extent with great significance for human health.

## Data Availability

The original contributions presented in the study are included in the article/Supplementary Material, further inquiries can be directed to the corresponding author.
