# Supplementary material for: The therapeutic efficacy of resveratrol for acute lung injury—A meta−analysis of preclinical trials
Source: Front Pharmacol. 2022 Aug 25;13:963245. doi: 10.3389/fphar.2022.963245 (PMC9453560; doi:10.3389/fphar.2022.963245)
Supplement: Supplementary file 1 [file DataSheet1.docx]

**S1 Appendix. Search strategy**

**PubMed**

**#1** (("Acute Lung Injury"[Mesh]) OR ("Respiratory Distress Syndrome"[Mesh]))

**#2** (((((((((((((((((Acute Lung Injuries[Title/Abstract]) OR (Lung Injuries, Acute[Title/Abstract])) OR (Lung Injury, Acute[Title/Abstract])) OR (Distress Syndrome, Respiratory[Title/Abstract])) OR (Distress Syndromes, Respiratory[Title/Abstract])) OR (Respiratory Distress Syndromes[Title/Abstract])) OR (Syndrome, Respiratory Distress[Title/Abstract])) OR (Shock Lung[Title/Abstract])) OR (Lung, Shock[Title/Abstract])) OR (Respiratory Distress Syndrome, Acute[Title/Abstract])) OR (Acute Respiratory Distress Syndrome[Title/Abstract])) OR (ARDS, Human[Title/Abstract])) OR (Human ARDS[Title/Abstract])) OR (Respiratory Distress Syndrome, Pediatric[Title/Abstract])) OR (Pediatric Respiratory Distress Syndrome[Title/Abstract])) OR (Respiratory Distress Syndrome, Adult[Title/Abstract])) OR (Adult Respiratory Distress Syndrome[Title/Abstract] ))

**#3**  #1 or #2

**#4** **("Resveratrol"[Mesh])**

**#5** **(((((((((((((((3,5,4'-Trihydroxystilbene[Title/Abstract]) OR (3,4',5-Trihydroxystilbene[Title/Abstract])) OR (3,4',5-Stilbenetriol[Title/Abstract])) OR (trans-Resveratrol-3-O-sulfate[Title/Abstract])) OR (trans Resveratrol 3 O sulfate[Title/Abstract])) OR (SRT 501[Title/Abstract])) OR (SRT501[Title/Abstract])) OR (SRT-501[Title/Abstract])) OR (cis-Resveratrol[Title/Abstract])) OR (cis Resveratrol[Title/Abstract])) OR (Resveratrol, (Z)-[Title/Abstract])) OR (trans-Resveratrol[Title/Abstract])) OR (trans Resveratrol[Title/Abstract])) OR (Resveratrol-3-sulfate[Title/Abstract])) OR (Resveratrol 3 sulfate[Title/Abstract]))**

**#6**  #4 or #5

**#7**  #3 and #6

**EMBASE**

**#1** 'acute lung injury'/exp OR 'adult respiratory distress syndrome'/exp

**#2** 'acute lung injuries':ab,ti OR 'lung injuries, acute':ab,ti OR 'lung injury, acute':ab,ti OR 'distress syndrome, respiratory':ab,ti OR 'distress syndromes, respiratory':ab,ti OR 'respiratory distress syndromes':ab,ti OR 'syndrome, respiratory distress':ab,ti OR 'shock lung':ab,ti OR 'lung, shock':ab,ti OR 'respiratory distress syndrome, acute':ab,ti OR 'acute respiratory distress syndrome':ab,ti OR 'ards, human':ab,ti OR 'human ards':ab,ti OR 'respiratory distress syndrome, pediatric':ab,ti OR 'pediatric respiratory distress syndrome':ab,ti OR 'respiratory distress syndrome, adult':ab,ti OR 'adult respiratory distress syndrome':ab,ti

**#3**  #1 or #2

**#4** 'resveratrol'/exp

**#5** **'3,5,4-trihydroxystilbene':ab,ti OR '3,4,5-stilbenetriol':ab,ti OR 'trans-resveratrol-3-o-sulfate':ab,ti OR 'trans resveratrol 3 o sulfate':ab,ti OR 'srt 501':ab,ti OR 'srt501':ab,ti OR 'srt-501':ab,ti OR 'cis-resveratrol':ab,ti OR 'cis resveratrol':ab,ti OR 'resveratrol, (z)-':ab,ti OR 'trans-resveratrol':ab,ti OR 'trans resveratrol':ab,ti OR 'resveratrol-3-sulfate':ab,ti OR 'resveratrol 3 sulfate':ab,ti**

**#6**  #4 or #5

**#7**  #3 and #6

**Web of Science**

**#1** TS= (Acute Lung Injury) OR TS= (Respiratory Distress Syndrome)

**#2** AB=(Acute Lung Injuries OR Lung Injuries, Acute OR Lung Injury, Acute OR Distress Syndrome, Respiratory OR Distress Syndromes, Respiratory OR Respiratory Distress Syndromes OR Syndrome, Respiratory Distress OR Shock Lung OR Lung, Shock OR Respiratory Distress Syndrome, Acute OR Acute Respiratory Distress Syndrome POR ARDS, Human OR Human ARDS OR Respiratory Distress Syndrome, Pediatric OR Pediatric Respiratory Distress Syndrome OR Respiratory Distress Syndrome, Adult OR Adult Respiratory Distress Syndrome )

**#3**  #1 or #2

**#4** **TS=(Resveratrol)**

**#5** **AB= (3,5,4'-Trihydroxystilbene OR 3,4',5-Trihydroxystilbene OR 3,4',5-Stilbenetriol OR trans-Resveratrol-3-O-sulfate OR trans Resveratrol 3 O sulfate OR SRT 501 OR SRT501 OR SRT-501 OR cis-Resveratrol OR cis Resveratrol OR Resveratrol, (Z)- OR trans-Resveratrol OR trans Resveratrol OR Resveratrol-3-sulfate OR Resveratrol 3 sulfate)**

**#6**  #4 or #5

**#7**  #3 and #6
